# Intraosseous mandibular schwannoma managed via submandibular approach: a case report with a review of previously published cases

**DOI:** 10.1093/jscr/rjae537

**Published:** 2024-08-28

**Authors:** Abdulaziz Alabdulkarim, Shaikha AlDukhail, Abdullah A Al Qurashi, Abdullatif Abuhaimed, Omar Alshameri, Abdulaziz Alghamdi, Alwaleed K Alammar, Abdulrahman Alsahabi

**Affiliations:** Plastic Surgery, Department of Surgery, College of Medicine, Prince Sattam Bin Abdulaziz University, Al Kharj, Saudi Arabia; Department of Preventive Dental Sciences, College of Dentistry, Princess Nourah bint Abdulrahman University, P.O. Box 84428, Riyadh 11671, Saudi Arabia; College of Medicine, King Saud bin Abdulaziz University for Health Sciences, Riyadh, Saudi Arabia; Oral and Maxillofacial Surgery Department, Ministry of National Guard, King Abdulaziz Medical City, Riyadh, Saudi Arabia; Oral and Maxillofacial Surgery Department, Ministry of National Guard, King Abdulaziz Medical City, Riyadh, Saudi Arabia; Oral and Maxillofacial Surgery Department, Ministry of National Guard, King Abdulaziz Medical City, Riyadh, Saudi Arabia; Plastic Surgery Department, Prince Sultan Medical City, Riyadh, Saudi Arabia; Plastic Surgery Department, Prince Sultan Medical City, Riyadh, Saudi Arabia

**Keywords:** schwannomas, neurilemmomas, mandible, osseous neoplasms, bone neoplasms, benign neoplasms, mandibular neoplasms

## Abstract

A 40-year-old female presented with right mandibular swelling. A panoramic radiograph showed a unilocular radiolucency from the mandibular angle to tooth #46. Biopsy confirmed a schwannoma. Surgical resection was performed via a submandibular approach with a reconstruction plate. Teeth 46 and 47 were extracted. Surgery was complication-free, and histopathology confirmed the tumor’s benign nature. The patient was discharged on the second postoperative day. At the 1-year follow-up, she had no paresthesia, normal mouth opening, and full mandibular motion. The reconstruction plate was intact. This case adds to the limited literature on intraosseous schwannomas, emphasizing early detection, thorough radiological assessment, and meticulous surgical planning.

## Introduction

Schwannomas, or neurilemmomas, are benign neoplasms originating from the peripheral nerve sheath, primarily composed of Schwann cells. While 25%–45% of schwannomas occur in the head and neck, their presence in the mandible’s bone structure is rare, constituting only 0.2% of all osseous neoplasms [1]. These tumors often grow slowly, causing localized swelling or pain, but can remain undetected until routine radiological screenings [2]. Radiographic features vary from unilocular to multilocular radiolucencies, complicating diagnosis [3]. Despite their benign nature, surgical intervention is required due to potential bone erosion, disturbance of dental structures, and the rare risk of malignant transformation [4].

This case report details a 40-year-old female with a mandibular schwannoma, supplemented by a table of similar cases for comparative insight. It highlights the importance of early detection, thorough radiological evaluation, and a tailored surgical approach for optimal outcomes.

## Case presentation

**Figure 1:**
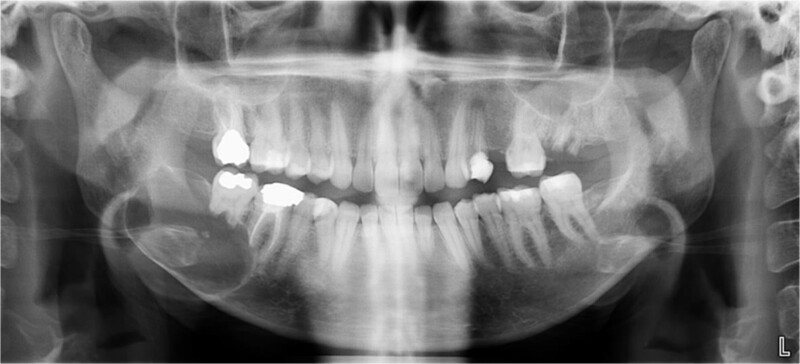
Panoramic radiograph showing a unilocular radiolucency extending from the right mandibular angle to the right first molar region.

**Figure 2:**
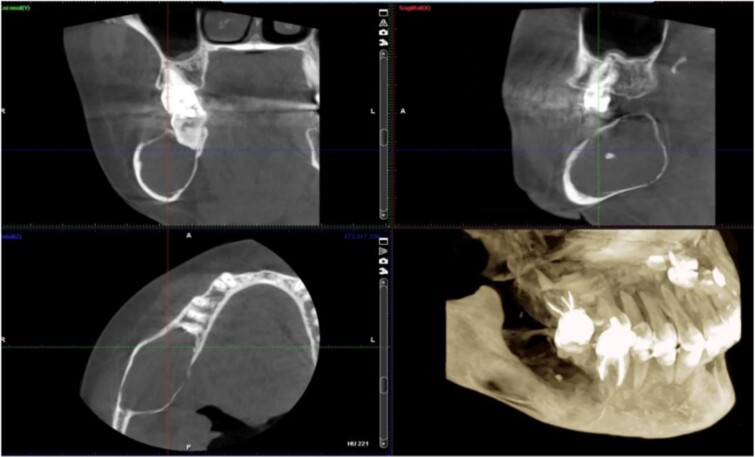
Panoramic radiograph highlighting the extent of the lesion from the mandibular angle to tooth #46.

**Figure 3:**
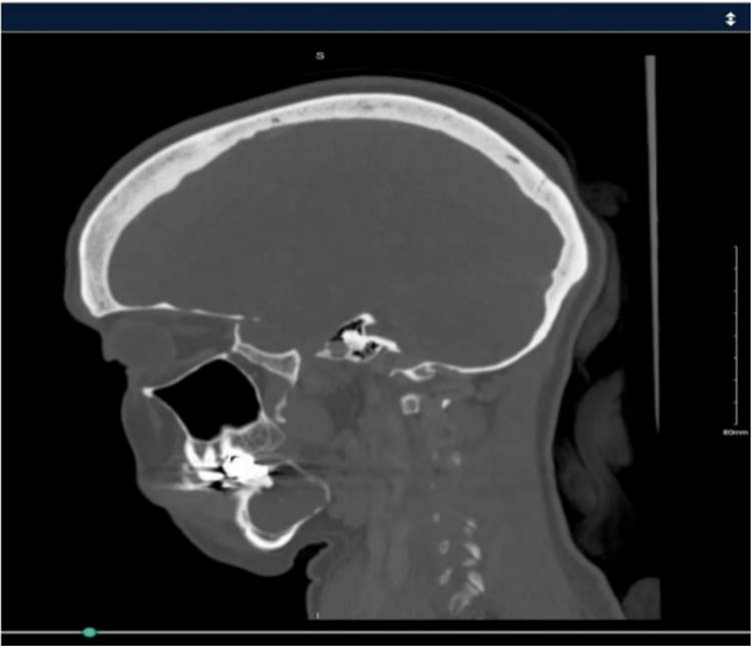
Intraoperative view of the surgical site showing the resection area and preservation of the submandibular gland and marginal mandibular branch of the facial nerve.

A 40-year-old woman presented with swelling on the right side of her mandible. She had completed orthodontic treatment 2 years earlier. A panoramic radiograph showed a unilocular radiolucency extending from the mandibular angle to the right first molar region (Figs 1–3).

**Figure 4:**
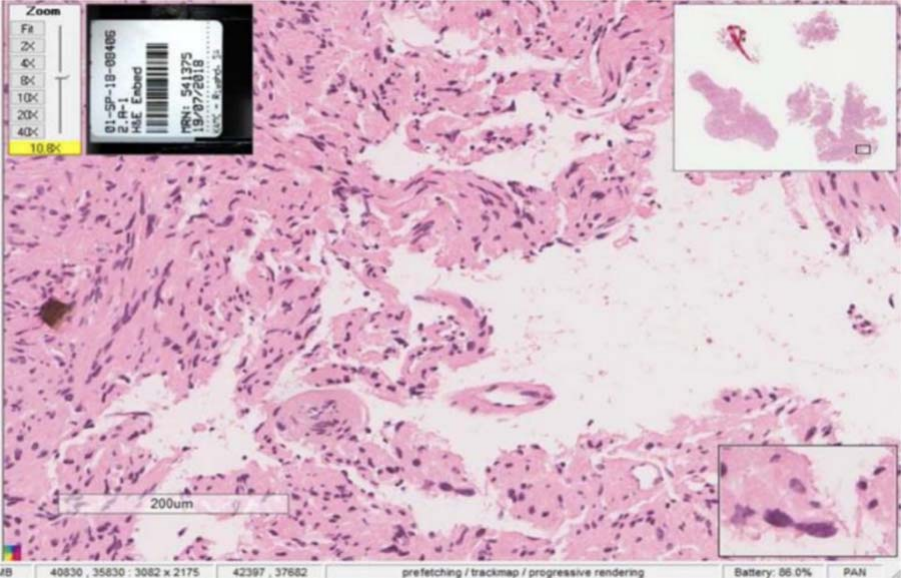
Histopathological examination showing Antoni A and Antoni B patterns with hyalinized Verocay bodies among spindle-shaped cells.

**Figure 5:**
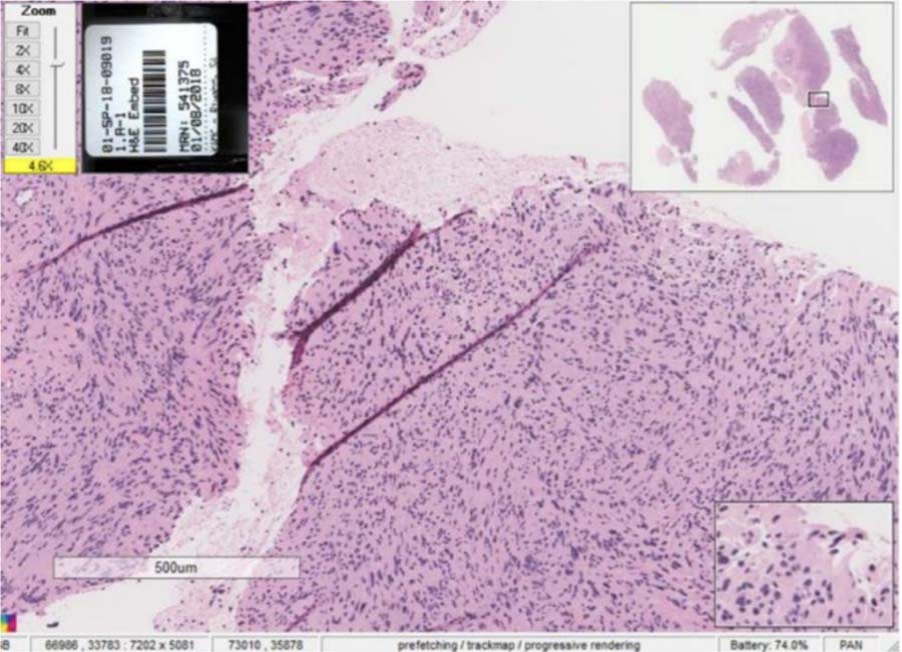
Close-up view of the histopathological slide confirming the diagnosis of schwannoma.

An incisional biopsy under local anesthesia revealed Antoni A and B patterns with hyalinized Verocay bodies among spindle-shaped cells, diagnosing the mass as a schwannoma (Figs 4 and 5).

Surgical resection was performed via a submandibular approach, with an incision from the anterior edge of the masseter to the mid-neck. The submandibular gland was preserved, and the marginal mandibular branch of the facial nerve was protected.

The tumor’s encroachment on the thin mandibular bone required the removal of the outer table for access and enucleation. Despite cortical breakthrough in the lingual table, the intact periosteum facilitated cancellous bone grafting. The lingual nerve remained unexposed.

An iliac block graft replaced the removed cortical layers, secured with a plating system to maintain mandibular integrity. The patient received intravenous antibiotics for 3 days postoperatively, without needing intermaxillary fixation. She was on a soft diet for 2 weeks, transitioning to a regular diet within 6 weeks.

**Figure 6:**
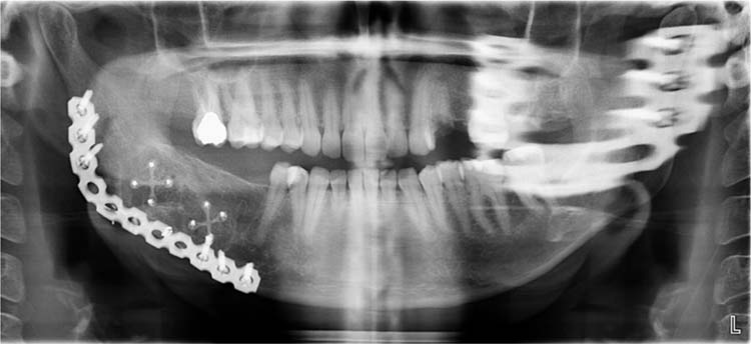
Postoperative panoramic radiograph demonstrating the reconstruction plate in place after tumor resection and bone grafting.

**Figure 7:**
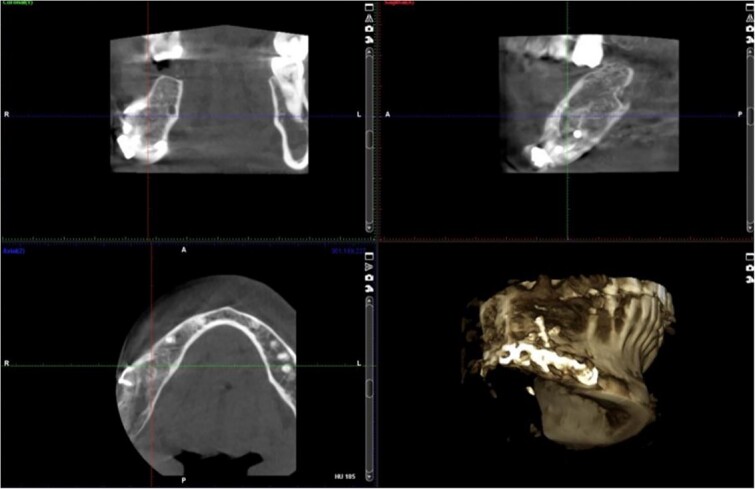
Follow-up panoramic radiograph showing stable reconstruction plate and no signs of recurrence one year postoperatively.

The excised specimen’s histopathology confirmed its benign nature. One year postoperatively, the patient showed no infection or inflammation, with optimal mouth opening and mandibular movement. The reconstruction plate remained stable and intact (Figs 6 and 7).

For comparative insights, see Table 1 for similar intraosseous schwannoma cases.

**Table 1:** Comprehensive overview of reported mandibular schwannoma cases to date.

**Case No.**	**Title**	**Summary**
1	Maxillary schwannoma–a case report of a rare tumor	A 22-year-old male presented with a rare case of maxillary sinus schwannoma extending to the nasal cavity and infratemporal fossa. The tumor was excised with a subtotal maxillectomy using Weber–Fergusson approach.
2	Imaging Features of Intraosseous Schwannoma: A Case Series and Review of the Literature	Over 17 years, 6 patients diagnosed with intraosseous schwannoma (IOS) were evaluated through imaging studies. IOSs, rare benign neoplasms, displayed characteristic imaging features, and are usually found in the lumbosacral region.
3	Surgical Management of Mandibular Intraosseous Schwannomas	Schwannomas of the mandible are rare tumors typically associated with the inferior alveolar nerve. The preferred treatments are surgical, with various methods available depending on the tumor size and location.
4	Intraosseous ancient schwannoma: a rare case in the mandible and a literature review	A 24-year-old male was diagnosed with an intraosseous ancient schwannoma in the mandible, initially misdiagnosed as carcinoma. The tumor’s unique features and management are discussed in light of the literature.
5	Unusual intramaxillary plexiform schwannoma	An extremely rare case of an intraosseous plexiform schwannoma located in the maxilla is presented. Schwannomas of this type have only been reported once before in the jaw bones, specifically the mandible.
6	Recurrent Ancient Intraosseous Neurilemmoma of Maxilla: A Rare Case Report	A distinctive case of a 38-year-old male with recurrent intraosseous ancient neurilemmoma in the maxilla is presented. The tumor had unique histological features and recurred in a relatively short time frame.
7	Central schwannoma of mandible	A 23-year-old female was diagnosed with a central schwannoma in the mandible. The tumor was treated with segmental resection and replaced with an autogenous iliac bone graft.
8	Intraosseous neurilemoma of the mandible with unusual multilocular presentation: a case report	A 12-year-old patient exhibited an intraosseous neurilemoma in the mandible with a rare multilocular presentation. The lesion had been present for at least 3 years prior to diagnosis, initially appearing as a unilocular radiolucency.
9	CT and MRI findings of intraosseous schwannoma of the mandible: a case report	Radiographical findings from CT and MRI scans of a rare intraosseous schwannoma in the mandible are presented. The lesion encased the mandibular canal and caused some bone cortex destruction, yet it maintained well-demarcated borders.
10	Intraosseous schwannoma of mandibular symphysis: case report	An 11-year-old boy was diagnosed with an intraosseous schwannoma located in the mandibular symphysis. The tumor was surgically excised, and no recurrence was observed over a 5-year follow-up.
11	Intraosseous schwannoma mimicking a periapical lesion on the adjacent tooth: case report	A 34-year-old female presented with an intraosseous schwannoma in the mandibular alveolar bone, which resembled an inflammatory periapical lesion. The lesion could be misdiagnosed as endodontic in origin, making histological examination essential for accurate diagnosis.
12	Multiple schwannomas of the facial nerve mimicking cervical lymphoma: a case report	Multiple schwannomas of the marginal mandibular branch of the facial nerve were identified in a Caucasian patient. It is the first documented case of its kind. The tumors were identified after the patient experienced sudden lower facial nerve palsy. Surgical intervention was carried out with nerve monitoring.
13	Central neurilemmoma (schwannoma) of the mandible. Case report	A 55-year-old Vietnamese woman had a central neurilemmoma (schwannoma) in the posterior mandible. Such presentations as a central bone tumor are rare, with fewer than 40 cases reported in the jaws. This benign neurogenic tumor’s incidence, clinical features, radiographic appearance, histology, and treatment methods were discussed.
14	Central neurilemmoma of the jaw in concurrence with radicular cyst: a case report	A 29-year-old woman experienced numbness on the right side of her lower lip for 3 months after undergoing endodontic therapy on her right mandibular first molar. Panoramic radiography showed a bilocular radiolucency in the mandible’s right body. An excisional biopsy confirmed it as a central neurilemmoma coexisting with an inflammatory apical dental (radicular) cyst. There was no recurrence in a 1-year follow-up.
15	Cellular schwannoma of the mandible: a case report	Schwannomas are benign tumors derived from the sheath cells surrounding myelinated nerve fibers, commonly found in the head and neck. Cellular schwannomas, however, differ from classic schwannomas due to their heightened cellularity, nuclear variations, lack of Verocay bodies, and often increased mitotic activity. These tumors typically appear in middle-aged individuals and are frequently found in specific body areas, such as the retroperitoneum and head and neck. Despite their resemblance to malignant tumors microscopically, they rarely recur and have not been reported to metastasize.

## Discussion

Schwannomas, or neurinomas, are benign neoplasms derived from Schwann cells of the peripheral nerve sheath [1]. Intraosseous schwannomas (IOS) in the mandible are rare, comprising only 0.2% of all osseous neoplasms [5]. These tumors, typically presenting in middle-aged adults, are slow-growing and often asymptomatic, leading to delayed diagnosis [4, 6].

In our case, the schwannoma was intramandibular, consistent with other reports. Surgical intervention involved a unicortical corticotomy, removing only the outer cortical plate to preserve mandibular integrity and nerve function, particularly the inferior alveolar nerve [7, 8]. This approach minimizes structural weakening and aligns with recent studies advocating for minimal invasiveness and precision [9].

We used X-shaped miniplates for reconstruction due to their biomechanical advantages, providing superior stability and strength [10, 11]. The right first molar and another compromised tooth were extracted to facilitate tumor removal and prevent infection or instability [12, 13].

A segment of the mandible’s thin outer table was removed to access the tumor, and an iliac block graft replaced the cortical layers, supported by X-shaped miniplates for stability [14]. This approach ensured complete tumor removal without exposing the lingual nerve.

Postsurgical management included 3 days of IV antibiotics, no need for intermaxillary fixation, and a soft diet for 2 weeks, transitioning to a regular diet within 6 weeks. Follow-ups confirmed optimal recovery and functional outcomes, with no infection or inflammation, and stable reconstruction plates [15].

Radiographic evidence showed expansile lytic lesions with sclerotic borders, consistent with IOS presentations [16]. Histology revealed Antoni A and B patterns, hyalinized Verocay bodies, and S-100 protein-positive cells, corroborating previous findings [17, 18].

Surgical management typically involves enucleation through a mucoperiosteal flap and a bone window, emphasizing thorough postoperative monitoring due to the risk of recurrence [19]. Imaging modalities such as CT and MRI are valuable but not mandatory [5, 19]. Our submandibular approach was effective and consistent with other studies [19, 20].

In conclusion, this case and the literature underscore the importance of early detection, comprehensive radiological assessment, and a meticulous surgical approach for managing mandibular schwannomas, ensuring optimal outcomes and improved patient quality of life.

## Conclusion

Given their rarity, IOS require a thorough understanding of their clinical, radiological, and histological features for timely diagnosis and appropriate treatment. Diligent postoperative monitoring is essential to detect potential recurrences from possibly incomplete surgical interventions. This case highlights the importance of comprehensive diagnostic assessments and strategic surgical planning in ensuring patient health and successful long-term outcomes. Additionally, our table summarizing previously reported cases provides a holistic understanding of this rare condition.
